# Triple negative breast cancers express receptors for LHRH and are potential therapeutic targets for cytotoxic LHRH-analogs, AEZS 108 and AEZS 125

**DOI:** 10.1186/1471-2407-14-847

**Published:** 2014-11-19

**Authors:** Stephan Seitz, Stefan Buchholz, Andrew Victor Schally, Florian Weber, Monika Klinkhammer-Schalke, Elisabeth C Inwald, Roberto Perez, Ferenc G Rick, Luca Szalontay, Florian Hohla, Sabine Segerer, Chui Wai Kwok, Olaf Ortmann, Jörg Bernhard Engel

**Affiliations:** Department of Gynecology and Obstetrics, University Medical Center Regensburg, 93053 Regensburg, Germany; Endocrine, Polypeptide and Cancer Institute, Veterans Affairs Medical Center and South Florida Veterans Affairs Foundation for Research and Education, Miami, FL 33125 USA; Department of Pathology and Medicine, Division of Hematology/Oncology and Endocrinology, Miller School of Medicine, University of Miami, Miami, USA; Department of Pathology, University Medical Center Regensburg, 93053 Regensburg, Germany; Tumor Center Regensburg e.V., University of Regensburg, Regensburg, Germany; Department of Urology, Herbert Wertheim College of Medicine, Florida International University, Miami, USA; Medicine with Haematology, Oncology, Rheumatology and Infectiology, Private Medical University of Salzburg, Salzburg, Austria; Endokrinologikum Hamburg, 22767 Hamburg, Germany; Depertment of Obsteterics and Gynecology, Medical University of Gießen, 35392 Gießen, Germany

**Keywords:** Targeted therapy, Triple negative breast cancer, LHRH- receptor, AEZS 108, AEZS 125

## Abstract

**Background:**

Triple negative breast cancer (TNBC) is a distinct subtype of breast cancer burdened with a dismal prognosis due to the lack of effective therapeutic agents. Receptors for LHRH (luteinizing hormone-releasing hormone) can be successfully targeted with AEZS-108 [AN-152], an analog of LHRH conjugated to doxorubicin. Our study evaluates the presence of this target LHRH receptor in human specimens of TNBC and investigates the efficacy and toxicity of AEZS-108 *in vivo.* We also studied *in vitro* activity of AEZS-125, a new LHRH analog conjugated with the highly potent natural compound, Disorazol Z.

**Methods:**

69 human surgical specimens of TNBC were investigated for LHRH-R expression by immunohistochemistry. Expression of LHRH-R in two TNBC cell lines was evaluated by real time RT-PCR. Cytotoxicity of AEZS-125 was evaluated by Cell Titer Blue cytoxicity assay. LHRH- receptor expression was silenced with an siRNA in both cell lines. For the *in vivo* experiments an athymic nude mice model xenotransplanted with the cell lines, MDA-MB-231 and HCC 1806, was used. The animals were randomised to three groups receiving solvent only (d 1, 7, 14, i.v.) for control, AEZS-108 (d 1, 7, 14, i.v.) or doxorubicin at an equimolar dose (d 1, 7, 14, i.v.).

**Results:**

In human clinical specimens of TNBC, expression of the LHRH-receptor was present in 49% (n = 69).

HCC 1806 and MDA-MB-231 TNBC cells expressed mRNA for the LHRH-receptor. Silencing of the LHRH-receptor significantly decreased the cytotoxic effect of AEZS-108. MDA-MB-231 and HCC 1806 tumors xenografted into nude mice were significantly inhibited by treatment with AEZS-108; doxorubicin at equimolar doses was ineffective.

As compared to AEZS 108, the Disorazol Z – LHRH conjugate, AEZS-125, demonstrated an increased cytotoxicity *in vitro* in HCC 1806 and MDA-MB-231 TNBC; this was diminished by receptor blockade with synthetic LHRH agonist (triptorelin) pretreatment.

**Conclusion:**

The current study confirms that LHRH-receptors are expressed by a significant proportion of TNBC and can be successfully used as homing sites for cytotoxic analogs of LHRH, such as AEZS-108 and AEZS-125.

## Background

The hypothesis of a ‘magic bullet’ that could specifically eradicate cancers was conceived in 1898 by Paul Ehrlich, but remained undeveloped for decades. Following the discovery that tumor cells express certain specific extra- or intracellular proteins, the concept of using receptor proteins as potential targets for “magic bullets” became applicable to tumor therapy [1].

Breast cancer is a heterogeneous disease that encompasses several distinct entities with different biological characteristics and clinical behaviors. Currently, breast cancer patients are treated by approaches based on various clinical parameters in conjunction with assessment of the status of sex steroid receptors (estrogen and progesterone receptors) and the overexpression of HER2. Although effective endocrinologically tailored therapies have been developed for patients with hormone receptor-positive or HER2-positive disease, at present chemotherapy is the only modality of systemic therapy for patients with triple-negative breast cancers.

The definition of triple-negative breast cancer (TNBC) refers to a group of tumors, which do not express receptors for estrogen or progesterone and which do not overexpress the HER2 receptor. Tumors belonging to this subgroup often are of the basal-like subtype, i.e. they express genes that are characteristic of basal epithelial cells. However, not all TNBC are basal-cell like tumors, therefore these two expressions are not used as synonyms. TNBCs show distinctive clinical features and account for 10–17% of all breast carcinomas [2, 3]. TNBCs tend to more frequently affect younger patients [4], are more prevalent in African Americans, [5] and are clinically more aggressive than tumors belonging to the other known clinical subgroups [2, 3, 6, 7]. As TNBCs do not express the potential therapeutic targets mentioned above (i.e. receptors for estrogen, progesterone or HER2) targeted therapy has not been possible and chemotherapy has been the only therapeutic option for these patients. Although TNBCs are sensitive to chemotherapy [2], the response rates are low, the prognosis remains poor. Thus, in patients with TNBC disease recurrence occurs earlier and most deaths occur in the first five years after diagnosis [3, 8]. These observations underline the importance of identifying specific therapeutic targets for this breast cancer subgroup.

Specific receptors for LHRH were originally detected in the pituitary gland, but were also described in healthy tissue of male and female reproductive organs. They expressed only at low levels or not at all by other, benign, tissues. Strikingly, these receptors have also been detected on a variety of human cancer cells, such as breast, prostatic, ovarian and endometrial, making them suitable targets for specific targeted tumor therapy [9–19]. Predicated on these findings, a new class of antitumor compounds based on LHRH has been developed for targeted chemotherapy. In this approach agonists or antagonists of LHRH are used as carriers to deliver cytotoxic agents directly to cancerous cells, thereby increasing the local concentration of the cytotoxic drug in the tumor tissue while sparing normal, non-cancerous cells from unnecessary damage [20]. In recent years, cytotoxic analogs of various peptides containing doxorubicin have been developed. AEZS-108 (also known as AN-152) is such a cytotoxic hybrid molecule and consists of doxorubicin linked to the LHRH agonist, [D-Lys6] LHRH [17, 19–21].

A pilot study, performed by our group, demonstrated, by immunohistochemistry, RT-PCR, and Western blot analysis, that LHRH receptors are expressed on TNBC tissues. However, only 17 tumor specimens were analysed in this study [22].

In the current study a larger TNBC specimen group is analyzed with respect to LHRH receptor expression and a possible correlation with clinical stage and histopathological parameters. Additionally, the efficacy and toxicity of cytotoxic LHRH analog, AEZS-108, is tested in two models of TNBC *in vivo*.

The LHRH receptor targeting concept offers the possibility of replacing doxorubicin with even more potent cytotoxics, but with the advantage of increasing anticancer activity without enhancing organ toxicity. Thus, doxorubicin in AEZS-108 was replaced by Disorazol Z which was isolated from myxo-bacteria and which has anti-proliferative activity in the pico to low nano-molar range [23]. The cytotoxic potency of AEZS-125 was confirmed in two TNBC models *in vitro* and its LHRH receptor targeting was confirmed by competition experiments with the LHRH agonist, triptorelin.

## Methods

### Peptides and cytotoxic radicals

Cytotoxic LHRH-conjugate, AEZS-108, was originally synthesized in our laboratory (AVS) by coupling one molecule of doxorubicin-14-O-hemiglutarate to the ϵ-amino group of the D-Lys side chain of the carrier peptide [D-Lys6] LHRH [17, 21]. The batch of AEZS-108 used for this work was provided by Aeterna-Zentaris. Cytotoxic doxorubicin hydrochloride was obtained from Chemex Export–import Gmbh (Vienna, Austria). Before intravenous (i.v.) injection, the compounds were dissolved in 5% (w/v) aqueous D-mannitol solution (Sigma, St Louis, MO).

AEZS-125 and Disorazol Z was kindly provided by Dr. Michael Teifel, Aeterna-Zentaris GmbH, Frankfurt, Germany.

### Cell lines

HCC 1806 and MDA-MB-231 triple negative human breast cancer cell lines were obtained from American Type Culture Collection (Bethesda, MD). HCC 1806 cells were grown in RPMI 1640 cell culture medium (ATCC Bethesda, MD) supplemented with 10% FBS and antibiotics in an 95% Air/5% CO_2_ atmosphere at 37°C. MDA-MB-231 cells were cultured in the Dubecco’s modified essential medium (DMEM) supplemented with 10% fetal bovine serum (FBS) and penicillin/streptomycin at 37**1** C and 5% CO2 atmosphere. Chemicals, unless stated otherwise, were purchased from Sigma (St. Louis, Missouri, USA).

### Screening of HCC1806 and MDA-MB231 cells for receptor expression

Cells of HCC1806 human TNBC were cultured in flat bottom tissue culture plates using adherent conditions. Cells were collected from adherent cultures using trypsin dissociation. Cells were counted using a hemocytometer and trypan blue exclusion assay. Approximately 1.0 × 10^6^ cells were centrifuged and used for RNA isolation.

RNA isolation was performed with the GE Illustra RNA isolation kit as recommended by the manufacturer. RNA was quantified using a nanodrop spectrophotometer and 100 ng used for the analysis of LHRH (also known as GnRH), LHRH-R (also known as GnRH-R), ESR, Her2, and PgR expression with the Bio-Rad One-Step RT-PCR with SYBR kit. (Table 1) All reactions were performed with the Bio-rad CFX real-time PCR system. Normalization of gene expression was conducted using the geometric mean of the relative quantities of actβ and GAPDH (δδCt method, appendix 1). Human pituitary RNA and human fibroblast RNA was used as positive controls for all reactions. Mouse skin RNA was used as negative control for all reactions since our primers were designed to strictly match only the human sequences.

**Table 1:** **Sequence information for the oligonucleotide primers used for real-time RT-PCR analysis**

Primer name	Accession Number	Prod Len	Prod Tm	5′-Sense Primer-3′	Position	5′-Anti-sense Primer-3′	Position
Control							
HS-rt-actB	NM_001101	89	72.1	CCCACTTCTCTCTAAGGA	1,516	CATTACATAATTTACACGAAAGC	1,604
HS-rt-GAPDHv2	NM_002046	114	74.5	TGAGAAGTATGACAACAGC	513	ATGAGTCCTTCCACGATA	626
LHRH							
HS-rt-LHRH	NM_000825	77	70.5	CCTTTGTGGAAGTTATGTATG	410	CAGACCTATCAAGAGTTCAA	486
HS-rt-LHRHR	NM_000406	75	70.7	GAATAACTATCCAGCACTCA	811	TTCAAATTGGGACCACTTA	885
HORMONE							
HS-rt-ESR1	NM_000125	98	71.8	TTAGCCAAATTCTGTCTC	2,088	CACTAAGAACTGAGCAAG	2,185
HS-rt-HER2	NM_004448	98	71.4	AGCAATGGTGTCAGTATC	4,435	CCTGGGTCTTTATTTCATCT	4,532
HS-rt-PgR	NM_000926	75	70.7	TTGGAAGGATGGCTATTAC	7,243	AAGGATAAGTATGGATGAGAG	7,317

Amplified target (amplicons) sequences were confirmed be sequencing.

The real-time RT-PCR program consisted of a 30 minute reverse-transcription at 52°C followed by a simultaneous reverse transcriptase inactivation and polymerase activation at 95°C for 10 minutes. Once the polymerase was activated, the samples were subjected to 40 cycles of 2-stage PCR following the sequence of denaturing at 95°C, 10 seconds and annealing/extension at 57°C, 15 seconds. Melting curve analysis confirmed that the real-time RT-PCR resulted in only one product for each reaction and in no primer dimerization.

PCR reaction products were electrophoresed on a 2% agarose gel using 60 V for 100 minutes. Loading buffer was used which contained a final concetration of 2X SYBR green I DNA binding dye for visualization of the resulting bands.

### Fluorescent labeling of LHRHR on HCC1806 and MDA-MB-231 cells

Cells, cultured on sterile coverslips were used for immunofluorescent analysis. Specimens were incubated in 3% H_2_O_2_ in methanol for 5 minutes. Coverslips were washed with PBS three times, permeabilized in 0.2% Triton-X in PBS for 10 minutes and blocked with 2% goat serum in PBS for 30 min. LHRHR antibody (1:100 dilution, abcam ab58561) was added in PBS for 1 h. This was followed by 3 washes with PBS. Anti-goat secondary antibody (Alexa Fluor 488; Jackson Immunoresearch) was also applied for 1 h and then wahsed 3 times. Primary antibodies were applied for 30 minutes and fluorescent secondary antibodies (green) for 20 minutes. Coverslips were mounted in Vectashield mounting medium containing DAPI for nuclear staining (Vector Laboratories). Images were acquired on a Nikon Eclipse Ti fluorescence microscope (Nikon Instruments). Samples were mounted using standard optically clear mount medium. Cells are contrasted with DAPI-stained nuclei (blue).

### *In vitro*cell proliferation assay

The anti-proliferation effects of the toxic agent, Disorazol-Z, and its LHRH conjugate, AEZS-125, were investigated in the TNBC cell lines HCC1806 and MDA-MB-231.

Cells were starved in 1% FBS containing DMEM/F12 two days before treatment with the LHRH analogs. They were then trypsinized and counted 24 hours before treatment. 7500 HCC1806 or 3000 MDA-MB-231 cells were seeded in each well of a 96-well microplate with 100 μl serum free DMEM/F12. Three cultures of each cell type were tested for each concentration and three replicates were done for each of these.

Stock solutions of the compounds were made according to the provider’s instructions and were stored in 10 μl aliquots at -20°C. On the day of treatment, 100 μM working solutions in serum and phenol red free DMEM/F12 medium (Gibco, Darmstadt, Germany) were prepared from the stock solutions. Twelve half-log dilutions were done to produce a series of working solutions with concentrations from 0.0001 μM to 100 μM. For each well of the 96-well microplates, the contained medium was changed to 150 μl serum and phenol red-free DMEM/F12 supplemented with different concentrations of the drugs, or with the DMSO, H_2_O or PBS used as the solvent for the drugs.

After 48 hours, a cell titer blue (CTB) assay was performed by addition of 15 μl CTB reagent (Promega, Mannheim, Germany) to each well. The MDA-MB-231 and cells HCC1806 were then incubated under growth conditions for 1 hours and 4 hour, respectively. The color change and intensity of the CTB reagent was quantified with the Wallac Victor™3 1420 Microlabel Counter (Perkin Elmer, Rodgau, Germany) at a wavelength of 530 nm. The measured absorbance is proportional to the number of viable cells. EC50 was determined by the GraphPad Prism software (GraphPad, La Jolla, CA, USA). Experiments were performed in triplicates and repeated at least thrice.

### LHRH receptor blocking experiments

To determine whether the anti-proliferative activity of the Disorazol-Z LHRH conjugate AEZS-125 was mediated by LHRH receptor, an LHRH receptor blocking and competition study was carried out.

HCC1806 and MDA-MB-231 cells were starved and seeded in 96-well plates as described. On the day of treatment, the cells were incubated with 100 μM triptorelin or its solvent control, 1% DMSO, at 37°C for 10 minutes. After 10 minutes, the cells were washed with PBS and incubated with 0 to 10 μM AEZS-125 for an additional 10 minutes. The cells were washed again and cultivated in 150 μl serum and phenol red free DMEM/F12 at 37°C with 5% CO_2_/95% air for 48 hours until accomplishing the CTB assay.

### Small interfering RNA gene silencing

Silencing of LHRH-R was accomplished by reverse transfection using the siPORT NeoFX Transfection Reagent and Silencer Select siRNA (Applied Biosystems). Cells were trypsinized immediately before silencing. Cell suspensions were centrifuged at 3000 × g for 10 minutes and the media removed. Cells were suspended to a density of 10^5^ cells/ml in fresh media containing 10% FBS and antibiotic. RNA (1 μM) was diluted 1:4 in opti-MEM and 100 μl combined with 100 μl of 1:10 NeoFX solution per well. Transfection complexes were allowed to form for 15 minutes at room temperature. In each well of a 48 well culture plate, 250 μl of cell suspension was combined with 50 μl of complexes and cultured at 37°C and 5% CO_2_ for 72 hours, replacing the medium and transfection complexes after this incubation period. Silenced cultures were treated with either 500nM or 1 μM AeZS-108 for 72 hours at which time the media was replaced and an MTS colorimetric assay was used to determine proliferation relative to the untreated controls.

### Animals

Five- to six-week-old female athymic nude mice (Ncr nu/nu) were obtained from the National Cancer Institute (NCI, Bethesda, MD). The animals were housed in sterile cages under laminar flow hoods in a temperature-controlled room with a 12-h light/12-h dark schedule. They were fed autoclaved chow and water *ad libitum*.

### *In vivo*experiments

Cells of each cell line, growing exponentially, were implanted into 5 female donor nude mice by subcutaneous injection of 3 × 10^6^ cells into each flank. Tumors resulting after 4 weeks of growth were aseptically dissected and mechanically minced. In all experiments, 3 mm^3^ pieces of tumor tissue were transplanted subcutaneously (s.c.) into each experimental animal by trocar. Tumor volume (length × width × height × 0.5236) and body weight were measured weekly.

At the end of each experiment, the mice were killed under anesthesia, the tumors were excised and weighed, and necropsy was performed. Tumor specimens were snap frozen and stored at – 70 C. All experiments were performed in accordance with the institutional guidelines for the welfare of animals in experiments.

In experiment 1, when the MDA-MB-231 tumors had reached a volume of approximately 100 mm^3^, the mice were divided into three experimental groups of 9–10 animals each; each group received the following series of 3 injections on days 1, 8 and 15 into the jugular vein: group 1, control, vehicle solution (5% mannitol), group 2, cytotoxic analog AEZS-108 (2.5 mmol/kg) at a dose equivalent to 1.45 mg/kg DOX, group 3, cytotoxic radical DOX at 1.45 mg/kg. The experiment was terminated on day 28.

In experiment 2, when HCC 1806 tumors had grown to a volume of approximately 100 mm^3^, mice were assigned to three experimental groups of 5–6 animals each; each group received the following series of 3 injections on days 1, 8 and 15 injection into the jugular vein: group 1, control, vehicle solution, group 2, cytotoxic analog AEZS-108 (2.5 mmol/kg) at a dose equivalent to 1.45 mg/kg DOX, group 3, cytotoxic radical DOX at 1.45 mg/kg. The experiment was terminated on day 28.

The Institutional Animal Care and Use Committee (Medical Research Service of the Veterans Affairs Department) reviewed the protocol for the animal experiments and gave full approval. All the procedures *in vivo* were in accordance with UKCCCR guidelines for the welfare of animals in experimental neoplasia.

### Human specimens and detection of LHRH receptors by immunohistochemistry and clinical data set

Tumor samples and data were collected at the Tumor Center Regensburg a high quality population-based regional cancer registry covering a population of more than 2.2 million people of the districts of Upper Palatinate and Lower Bavaria and the University of Regensburg (Department of Gynecology and Obstetrics, Department of Pathology) following institutional guidelines and approval from the ethics committee of the University of Regensburg. Written informed consent for sample collection was obtained from all patients.

For immunohistochemistry, sections (4 to 5 μm thick) of tissue microarrays with probes of a total of 69 patients with confirmed TNBC were incubated with an antibody against LHRH receptor (Anti-GnRHR antibody A9E4, Abcam, UK) after previous antigen retrieval (3-min passages in a microwave oven at 750 watts in 10 mmol/l citrate buffer pH 6.0) at a dilution of 1:1500 for 30 min at room temperature. After drying overnight at 37°C, the EnVision combined peroxidase/diaminobenzidine detection system (Dako, Germany) was applied for visualization.

The available clinical data set was evaluated by grade, tumor size, and nodal status according to the WHO/TNM classification system and the histological subtype.

### Statistical analysis

For statistical analysis, Student’s two tailed *t*-test was used. A p value of less than 0.05 was considered as significant.

## Results

### Screening of HCC1806 and MDA-MB231 cells for receptor expression

**Figure 1:**
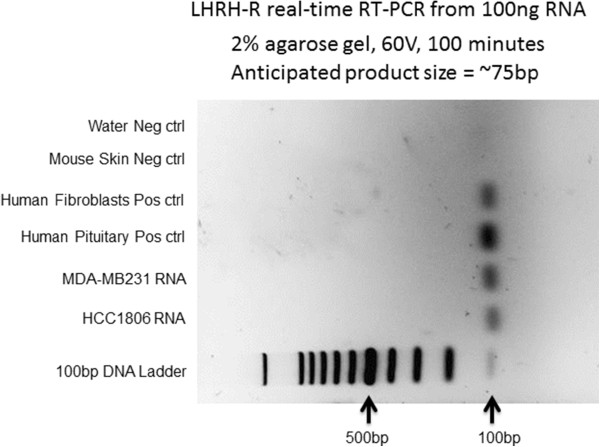
**PCR reaction products were electrophoresed on a 2% agarose gel using 60 V for 100 minutes.** Real-time one step RT-PCR analysis indicates that both HCC1806 and MDA-MB231 express LHRH-R. Human fibroblasts and human pituitary RNA were used as positive controls and mouse skin RNA was used as a species specificity control.

**Figure 2:**
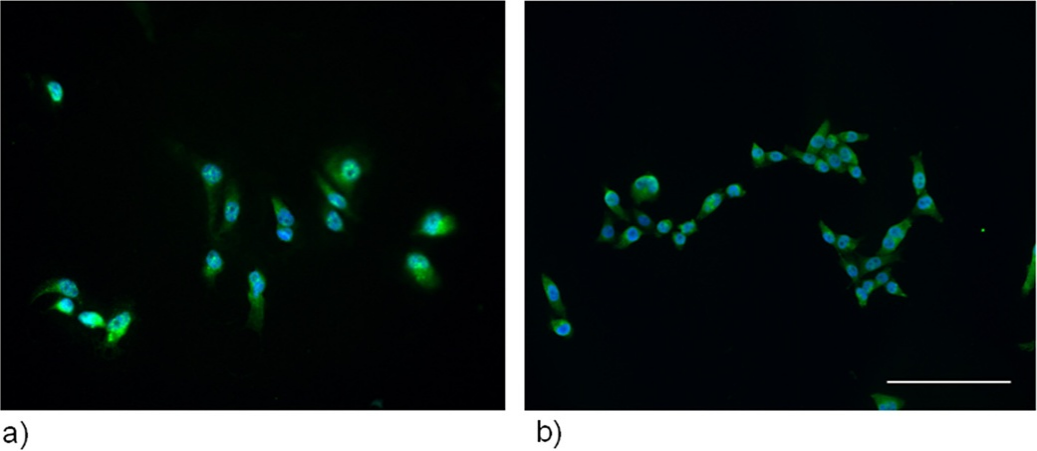
**Fluorescent micrograph of MDA-MB-231 (a) and HCC1806 (b) cells at 20x magnification.** Image shows blue DAPI-stained nuclei contrasting green labeled LHRH receptors.

HCC1806 and MDA-MB231 cells were found to express LHRH-R but not LHRH (Figure 1). Additionally, our analysis confirms that both cell-lines are TNBC and do not express ER nor PgR and do not overexpress Her2. Additionally, LHRH-receptors were demonstrated by fluorescent labeling on HCC-1806 and MDA-MB-231 cells (Figure 2 a,b).

### Inhibition of TNBC cell proliferation by the LHRH conjugate AEZS-125

**Figure 3:**
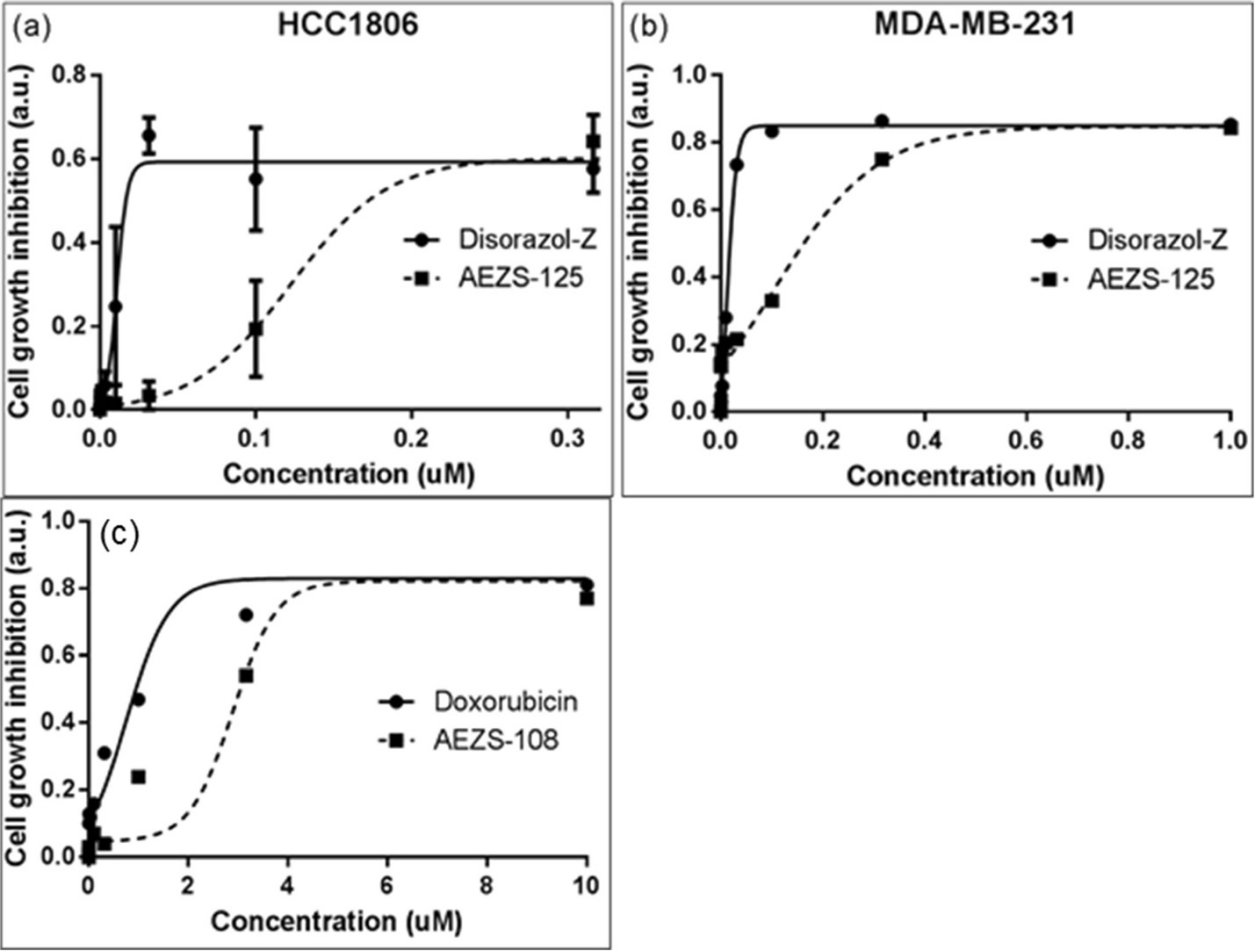
**Cytoxic effects of Disorazol-Z and its LHRH conjugate AEZS-125 in (a) HCC1806 and (b) MDA-MB-231; Doxorubicin and and its cytotoxic conjugate in MDA-MB-231 cells (c) as evaluated by CTB assay.**

**Figure 4:**
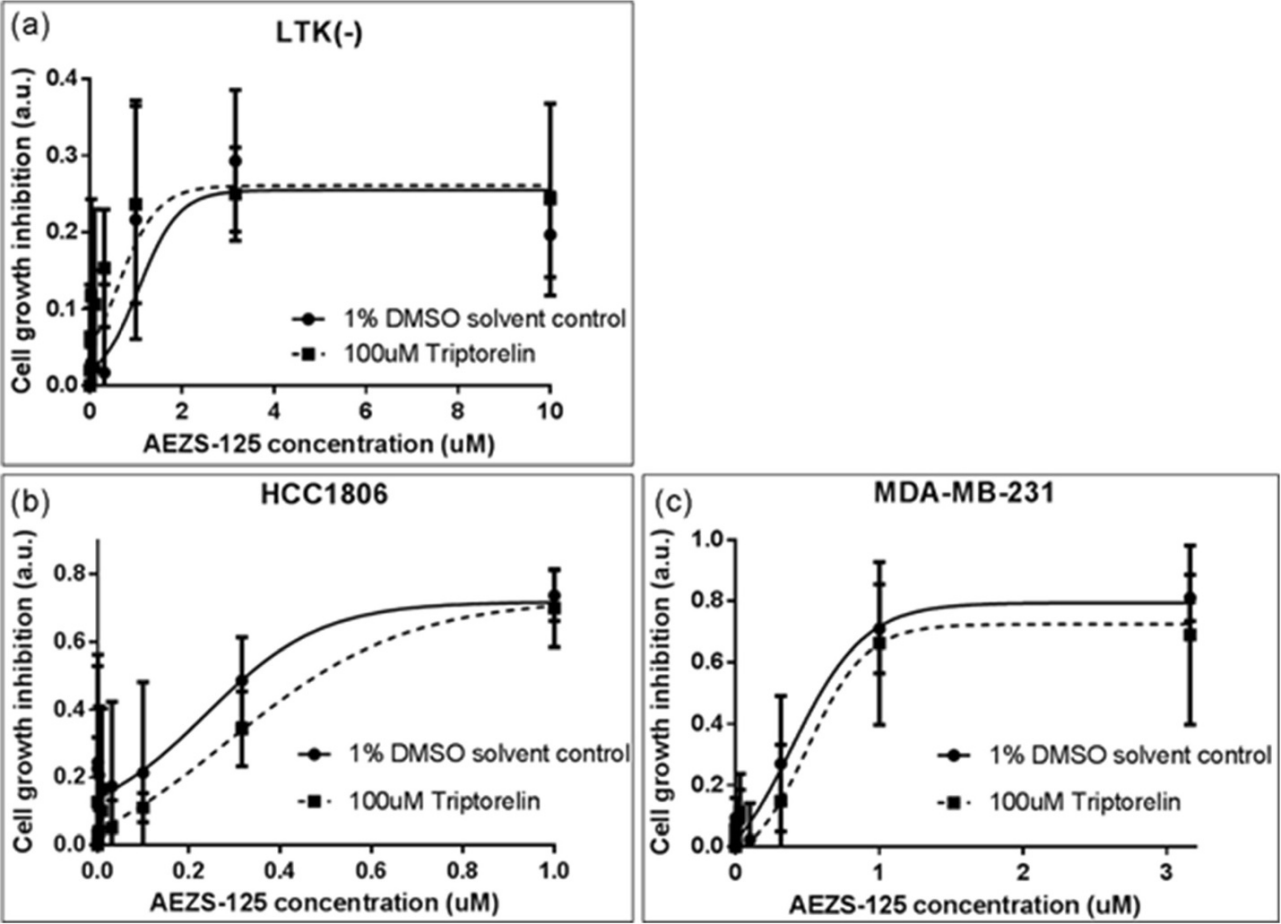
**Cytotoxic effects of AEZS-125 with and without pretreatment with LHRH agonist triptorelin, in LHRH- receptor positive TNBC cells MDA-MB-231 (c) and HCC 1806 (b) and LTK (-) (a) cells the last of which do not express receptors for LHRH.**

AEZS-125 is an LHRH conjugate with the cytotoxic drug, Disorazol-Z, and was found to be potent in inhibiting cell proliferation in TNBC cells (Figure 3 a,b). Disorazol-Z inhibited cell proliferation at low nanomolar concentrations. As expected, the hybrid cytotoxic compound AEZS-125 displayed lower cytotoxic effect *in vitro* than Disorazol, being the larger molecule. Doxorubicin and its cytotoxic conjugate, AEZS-108, also displayed significant cytoxicity in MDA-MB-231 cells (Figure 3c). As expected in an *in vitro* assay, the smaller molecule doxorubin was more cytotoxic than the conjugate. With an EC 50 in the low micromolar range AEZS-108 is the weaker cytotoxic agent as compared to AEZS-125. In order to show receptor mediated uptake of AEZS-125, LHRH- receptor blocking experiments with the LHRH analog, triptorelin, were performed (Figure 4).

### LHRH receptor mediated anti-proliferation activities of AEZS-125

In the control cell line LTK (-), which does not express any LHRH receptor, no reduction of the anti-proliferation activity of AEZS-125 was detected when the cells were pretreated with 100 μM triptorelin (Figure 2). This finding illustrated that triptorelin does not have any competitive effect with AEZS-125 in the absence of the LHRH receptor. In other words, the cell growth inhibitory effect of AEZS-125 observed with LTK (-) cells was LHRH receptor independent. On the other hand, the anti-proliferation effects of AEZS-125 in triptorelin pretreated HCC1806 and MDA-MB-231 cells was diminished (Figure 4, Table 2), but at a non-significant level.

**Table 2:** **EC50 values in the LHRH receptor expressing HCC1806 and MDA-MB-231 cells and in LHRH-receptor negative LTK (-) cells subsequent to coincubation with AEZS-125 with and without pretreatment with 100 μM triptorelin**

10-minute pretreatment	10-minute treatment	EC50 of AEZS-125 (nM)		
		LTK (-)	HCC1806	MDA-MB-231
1% DMSO solvent control	AEZS-125	1070.0 ± 351.1	238.1 ± 194.6	392.0 ± 136.6
		(n = 3)	(n = 3)	(n = 4)
100 μM Triptorelin	AEZS-125	613.8 ± 217.4	282.7 ± 151.2	499.0 ± 140.1
		(n = 3)	(n = 3)	(n = 4)

### Gene silencing of LHRH-R with small interfering RNA to determine the targeting ability of AEZS-108

Gene silencing with siRNA was performed in order to determine if the inhibitory activity of AEZS-108 is dependent on the expression of LHRH-R. Cultures were silenced with siRNA for LHRH-R for 72 hours at which time they were treated with either 500nM or 1 μM AeZS-108.

**Figure 5:**
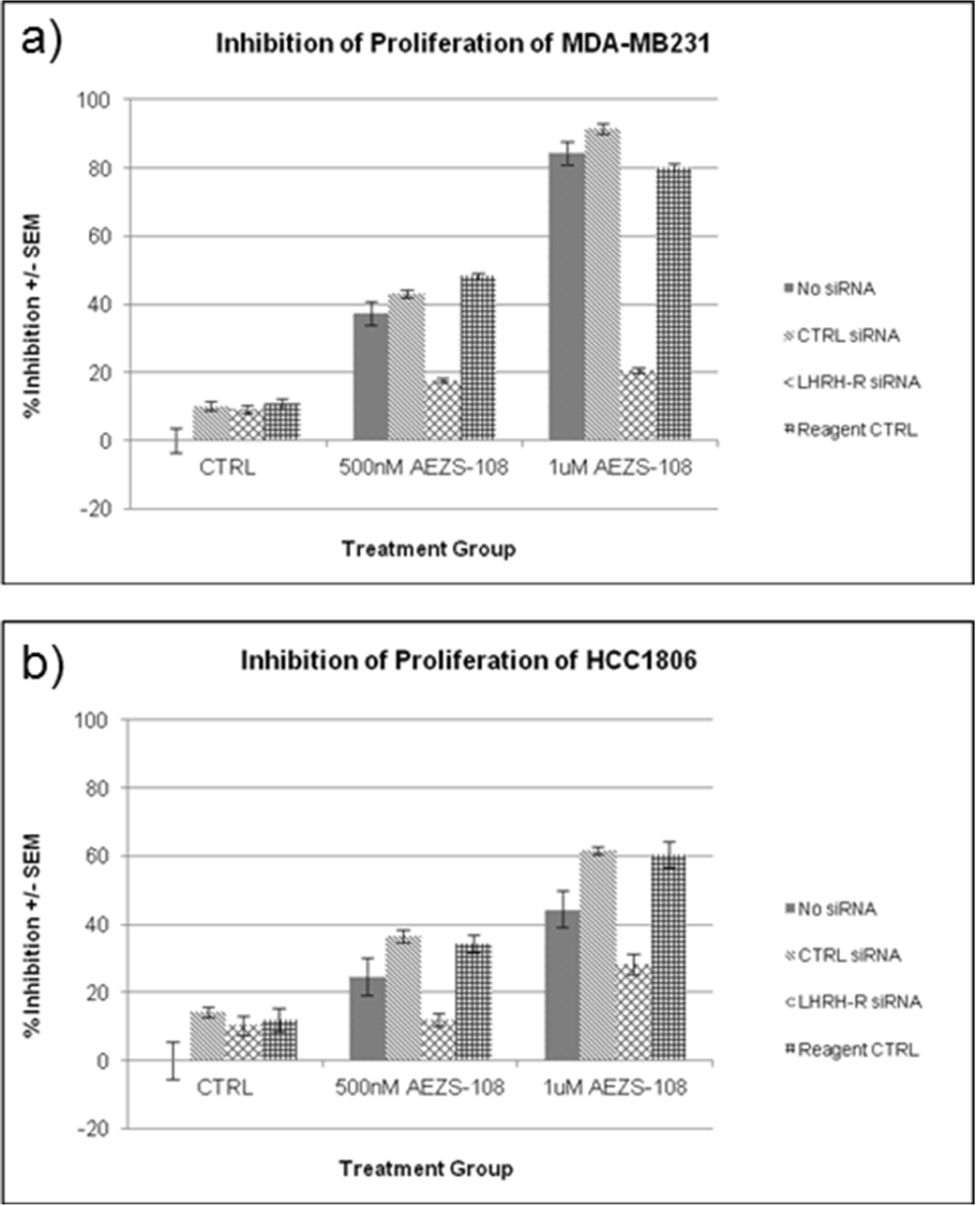
**Colorimetric determination of the proliferation of MDA-MB231 (a) and HCC 1806 (b).** Cultures were silenced for 72 hours and treated with AeZS-108 for an additional 72 hours. Silencing of LHRH-R significantly reduced the inhibitory activity of AEZS-108 in both ell lines (P <0.001, N = 12).

Treatment of MDA-MB231 breast cancer cells with AEZS-108 resulted in 37% and 84% less proliferation in the 500nM and 1 μM groups, respectively. Transfection of cells with scrambled human siRNA resulted in proliferation approximately equal to the controls. Likewise, treatment of the cells with only the transfection reagent resulted in proliferation approximately equal to the controls. Treatment of LHRH-R silenced cells of MDA-MB231 resulted in significantly less proliferation than any of the control groups (P < 0.001) (Figure 5a).

Treatment of HCC1806 breast cancer cells with AEZS-108 resulted in 50% and 37% less proliferation in the 500 nM and 1 μM groups, respectively. Transfection of cells with scrambled human siRNA resulted in proliferation approximately equal to the controls. Likewise, treatment of the cells with only the transfection reagent resulted in proliferation approximately equal to the controls. Treatment of LHRH-R silenced cells of HCC1806 resulted in significantly less proliferation than any of the control groups (P < 0.001) (Figure 5b).

### Effects of treatment with AEZS 108 on tumor growth *in vivo*

**Figure 6:**
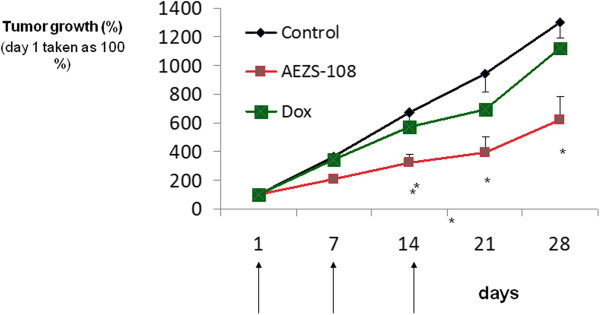
**Effects of targeted cytotoxic LHRH analog, AEZS-108, at doses equivalent to 1.45 mg/kg DOX and the radical DOX (administered i.v. on days 1, 7, 14) at equimolar doses on the growth of MDA-MB-231 TNBC xenografted into nude mice (mean ± se).** (* = p < 0.05 two sided Student’s *t*-test).

In the first experiment, 3 injections of cytotoxic LHRH analog, AEZS-108, equivalent to 1.45 mg/kg of DOXxHCl, significantly inhibited the growth of MDA-MB-231 human TNBC after 14 days compared with the control group (p < 0.05) and the group treated with equimolar doses of DOX alone (p < 0.05). The inhibitory effect of AEZS-108 remained significantly different from controls and DOX until the end of the study on day 28. Twenty-eight days after the injection of AN-152, tumor volume was reduced by 59% (p < 0.05) compared to the control group. An equimolar dose of the cytotoxic radical, DOX alone, had no significant growth inhibiting effects (Figure 6).

**Figure 7:**
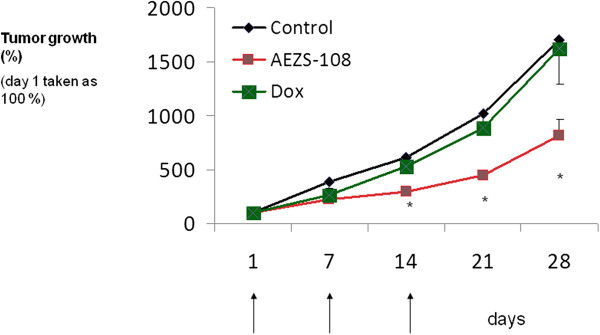
**Effects of targeted cytotoxic LHRH analog, AEZS-108, at doses equivalent to 1.45 mg/kg DOX and the radical DOX (administered i.v. on days 1, 7, 14) at equimolar doses on the growth HCC-1806 human TNBC (mean ± se).** (* = p < 0.05 two sided Student’s *t*-test).

In the second experiment, administration of 3 doses of cytotoxic LHRH-analog AEZS-108 equivalent to 1.45 mg/kg of DOXxHCl, significantly suppressed the proliferation (p < 0.05) of HCC-1806 human TNBC. Tumor volumes were significantly lower from treatment day 15 until the end of the experiment (p < 0.05). Twenty-eight days after the administration of AN-152, tumor volume was reduced by 52% (p < 0.05). An equimolar dose of the cytotoxic radical, DOX alone, had no significant effects on any tumor growth parameters (Figure 7).

### Immunohistochemistry and clinical data set

**Figure 8:**
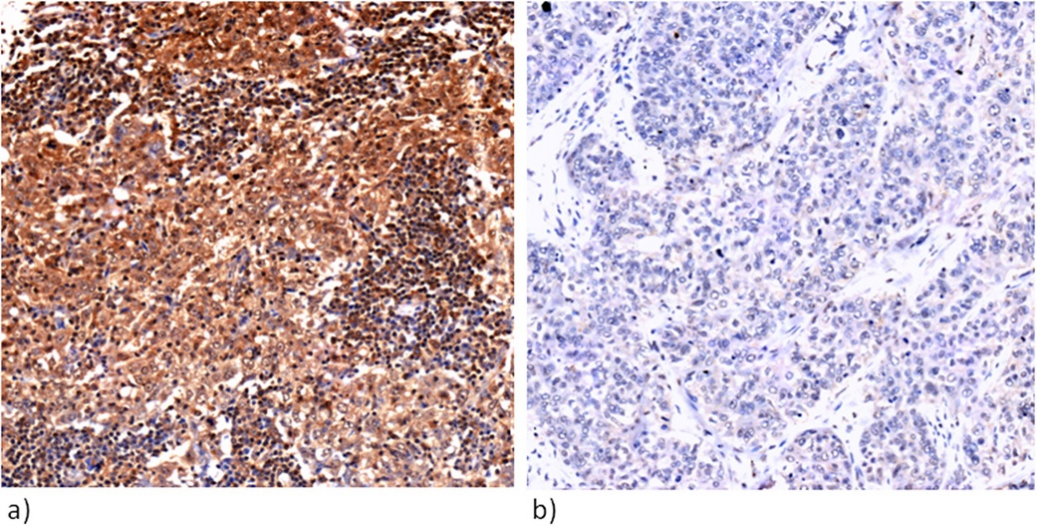
**Immunohistochemical evaluation of LHRH receptor expression: positive cytoplasmic staining reaction (a) and negative staining reaction (b) in triple negative human breast cancer samples (20×).**

The expression of the LHRH-R by immunohistochemistry in human triple negative breast tumor samples (n = 69) was detected in 49% (n = 34) (Figure 8). There was no association with grade, tumor size, nodal status or histological subtype and expression of the LHRH-R (Table 3).

**Table 3:** **LHRH-receptor expression of human specimens of TNBC**

	LHRH-R negative samples		LHRH-R positive samples	
	absolute	percent	absolute	percent
**T**				
T1	14	40	12	35
T2	17	49	18	53
T3	2	6	1	3
T4	0	0	2	5,9
Unknown	2	5,7	1	2,9
**N**				
+	11	31	7	21
-	18	51	19	56
unknown	7	20	8	23
**Grading**				
G1	0	0	1	3
G2	10	29	7	21
G3	25	71	26	77
**Histology**				
invasive ductal	29	82,8	28	82,3
invasive lobular	1	2,9	0	0
medullary	5	14,3	6	17,6

The LHRH-receptor positive and negative patient groups are descriptively compared with respect to size, nodal status, grading and histology of the tumors.

## Discussion

In the current study, which analysed the largest patient group to date, thirty-four out of 69 TNBC patients (49%) were positive for tumoral LHRH receptors. For the first time an attempt was made to correlate LHRH-receptor status with tumor stage and grade, lymph node status and histology of the tumor. However, no positive correlation was observed. The patient group was too small and the follow-up time too short to draw any conclusion on whether LHRH receptor status may be of prognostic use in TNBC.

After showing that LHRH receptors are expressed by both HCC 1906 and MDA-MB-231 cell lines, we also demonstrate that LHRH- receptor silencing by siRNA significantly decreases the cytotoxicity of AEZS-108, thus providing strong evidence for the receptor mediated effect of AEZS-108. Accordingly, in our *in vivo* studies in these LHRH-receptor positive models of human TNBC, three injections of AEZS-108 at doses equivalent to doxorubicin at 1.45 mg/kg significantly suppressed tumor growth from day 14 of treatment until the end of the experiment. Unconjugated doxorubicin at equimolar doses did not show any anti-tumor effect at all. Thus, we showed that tumoral LHRH-receptors in TNBC can be successfully targeted with AEZS-108, thus dramatically increasing the anti-tumor effect of doxorubicin.

In the current study it is also shown, for the first time, that the novel cytotoxic hybrid molecule AEZS-125, which is a conjugation of Disorazol-Z to D-Lys6-LHRH, induces strong cytotoxicity in TNBC cells. Disorazol-Z is an inhibitor of the mitotic spindle and induces cytotoxic effects in tumor cells at concentrations in the pico - to low nanomolar range [21]. Being several hundred times more potent than doxorubicin, it is therefore an ideal candidate to use for targeted chemotherapy. The marginal decrease of the EC 50 after blockade of the LHRH receptors, which does not occur in LHRH-receptor negative cells, suggests a receptor mediated uptake of AEZS-125, similar to the one already demonstrated for AEZS-108 [22]**.** However, as it is difficult to conclusively demonstrate receptor targeting *in vitro, in vivo* confirmation of targeting is mandatory and animal experiments with AEZS-125 in TNBC are already underway.

LHRH receptors have been found in >50% of human breast cancer specimens in a non- selected patient cohort which included ER positive, PR positive, HER2-neu overexpressing cancers as well as TNBC [20, 23]. AEZS-108 has already been tested in nude mice bearing xenografts of various human breast cancer lines including the LHRH receptor positive and doxorubicin-resistant human MX-1 breast cancer cell line. AEZS-108 significantly inhibited the growth of these MX-1 cells while the unconjugated doxorubicin was ineffective. The expression of mRNA for HER-2 and HER-3 and the levels of HER-2 and HER-3 proteins was also significantly reduced by the treatment with AEZS-108 [24]. Toxic side effects, such as leukopenia, were less pronounced in animals which had been treated with AEZS-108 compared to those treated with unconjugated doxorubicin [25].

Triple-negative breast cancer represents a subgroup of breast cancers burdened with a dismal prognosis due to the lack of specific therapies. In two recent studies in smaller patient groups LHRH receptors were detected in about 75% of human specimens [26]. Treatment of triple-negative, LHRH receptor positive MDA-MB-231, HCC1806 and HCC1937 human breast cancer cells with AEZS-108 resulted in apoptotic cell death as reflected by caspase-3 cleavage. The antitumor effects were confirmed *in vivo*, as AEZS-108 significantly inhibited the growth of the triple-negative breast cancers, HCC1806 and MDA-MB-231, xenografted into nude mice, without any apparent toxic side effects [1]

Due to good *in vivo* results in several other tumors, AEZS-108 has already been tested in Phase I and II studies in advanced ovarian and endometrial cancers [27]. In the phase I study the calculated t_1/2_ and clearance of AEZS-108 were approximately 2 h and 1 l/min m^2^, respectively [28]. At the dose levels of 160 and 267 mg/m2, average C_max_ values of DOX ranged from 600 to 700 ng/ml. As expected, average C_max_ and AUC of DOX were closely correlated to the AEZS-108 levels. In the first Phase II study, which was performed in collaboration with the German Gynecological Oncology Group (AGO), 43 patients with taxane-pretreated platinum-resistant LHRH receptor-positive ovarian cancer were included (). Partial remission in 5 patients (11.6%) and disease stabilization in 14 patients (32.6%) for > 12 weeks was achieved. Median time to progression was determined to be 3.5 months and median overall survival was 15 months [29].

In the second Phase II study 43 patients with histologically confirmed, LHRH-R positive, advanced (FIGO III or IV) or recurrent endometrial cancer were included [29]. Responses, confirmed by independent review, included 2 patients with complete response (CR; 5.1%), 10 patients with partial response (PR; 25.6%), and 17 patients with stable disease (SD; 43.6%). Based on those data, an overall response rate (ORR = CR + PR) of 30.8% and a clinical benefit rate (CBR = CR + PR + SD) of 74.4% can be estimated. Median time to progression (TTP) and overall survival (OS) were 7 months and 14.3 months, respectively. Responses were also achieved in patients with prior chemotherapy, 1 CR, 1 PR and 2 SDs in 8 patients who had been pretreated with platinum/taxane regimens [30].

In nude mice models AEZS-108 displayed weaker toxic side effects than equimolar doses of DOX. In particular no apparent toxic side effects to the pituitary, the heart, or other organs were observed. This excellent safety profile was further enhanced in pharmacologic safety studies evaluating the effects of AEZS-108 on respiratory and cardiovascular parameters in the dog, as well as in the Irwin and Rotarod test and in a hexobarbital interaction study. In these studies no test-item related effects were observed. In the cardiovascular safety study in beagle dogs, no evidence of QT prolongation was seen at any administered dose of AEZS-108. No adverse findings were observed in a local tolerability study in rabbits after intravenous and intra-arterial infusions of AEZS-108. Perivascular application of AEZS-108 induced moderate local inflammatory reactions. Superior tolerability of AEZS-108 as compared to DOX was further confirmed in acute and subchronic toxicity studies in mice, rats and dogs, respectively. In contrast to DOX, where lymphohistiocytic myocarditis with intramuscular fibrosis was observed, AEZS-108 did not induce any cardiotoxicity [22].

Accordingly, in the phase I and both phase II studies, there was no evidence of cardiotoxicity in serial controls of LVEF. As the pituitary has receptors for LHRH, pituitary toxicity of AEZS-108 was evaluated in the phase I study. No relevant effect of AEZS-108 on cortisol levels was observed in the ACTH stimulation test. Similarly, there was no effect of AEZS-108 on baseline serum levels of TSH, T3, and T4 and on the increase in TSH 30 min after stimulation with 200 μg TRH. Thus, at doses of 267 mg/m^2^ AES 108 has a favorable safety profile with manageable toxicity [28–30].

This reduction in toxicity during treatment with AEZS-108, compared to that with free doxorubicin, is likely due to the homing action of AEZS-108 to cells expressing LHRH receptors on their cell membrane. In contrast, free doxorubicin enters the cells by surface diffusion and accumulates in the nucleus independently of the presence of LHRH receptors on the cell surface.

## Conclusion

In conclusion, the current study shows LHRH receptor expression in 50% of human specimens of TNBC. This is the largest patient group so far analyzed. LHRH receptor expression did not correlate, however, with known prognostic factors, such as tumor stage, grade, or nodal status. *In vivo* studies with these two human breast cancer cell lines confirm that LHRH receptors on TNBC can be successfully targeted with the cytotoxic LHRH analog, AEZS 108. Previous work by our group [26], the study of *Foest et al.*[1], and the results of the current study, were the basis for the initiation of a Phase II trial which evaluates treatment with AEZS -125 in patients with advanced or metastatic LHRH receptor positive TNBC, and began patientrecruitment in January 2013.
